# *Helicobacter pylori* Virulence Factor Cytotoxin-Associated Gene A (CagA)-Mediated Gastric Pathogenicity

**DOI:** 10.3390/ijms21197430

**Published:** 2020-10-08

**Authors:** Shamshul Ansari, Yoshio Yamaoka

**Affiliations:** 1 Department of Microbiology, Chitwan Medical College, Bharatpur 44200, Nepal; ansari.shamshul@cmc.edu.np; 2 Department of Environmental and Preventive Medicine, Oita University Faculty of Medicine, Yufu, Oita 879-5593, Japan; 3 Global Oita Medical Advanced Research Center for Health (GO-MARCH), Yufu, Oita 879-5593, Japan; 4 Department of Medicine, Gastroenterology and Hepatology Section, Baylor College of Medicine, Houston, TX 77030, USA; 5 Borneo Medical and Health Research Centre, Universiti Malaysia Sabah, Kota Kinabalu, Sabah 88400, Malaysia

**Keywords:** *H. pylori*, virulence factor, cag pathogenicity island, CagA, gastric cancer

## Abstract

*Helicobacter pylori* causes persistent infection in the gastric epithelium of more than half of the world’s population, leading to the development of severe complications such as peptic ulcer diseases, gastric cancer, and gastric mucosa-associated lymphoid tissue (MALT) lymphoma. Several virulence factors, including cytotoxin-associated gene A (CagA), which is translocated into the gastric epithelium via the type 4 secretory system (T4SS), have been indicated to play a vital role in disease development. Although infection with strains harboring the East Asian type of CagA possessing the EPIYA-A, -B, and -D sequences has been found to potentiate cell proliferation and disease pathogenicity, the exact mechanism of CagA involvement in disease severity still remains to be elucidated. Therefore, we discuss the possible role of CagA in gastric pathogenicity.

## 1. Introduction

*Helicobacter pylori* is the most common bacterium, which colonizes the gastric epithelium of more than 50% of the world’s population [1,2]. Geographic variation, socio-economic status, urbanization level, and poor sanitation during childhood have been found to affect the infection prevalence variation between countries, with the highest prevalence in Asian, Middle American, and South American countries [2,3]. The global prevalence has been estimated to be 44.3%, whereas the highest prevalence of 89.7% and the lowest prevalence of 8.9% have been found in Nigeria and Yemen, respectively [1].

Although the exact route of transmission of *H. pylori* is still unclear, the epidemiological evidence supports its oral–oral or fecal–oral transmission from person to person, particularly among those in close contact, such as between mother and child, with a greater chance of infection transmission in childhood [4,5,6]. Yokota et al. conducted a study using multi-locus sequence typing and random amplified polymorphic DNA fingerprinting, and they revealed that the transmission of *H. pylori* occurs from mother to child after the first year of life [7]. Once *H. pylori* is transmitted to the gastric lumen, a permanent infection is established in the stomach, and if left untreated, leads to several gastro-duodenal conditions such as chronic gastritis, gastric ulcer, duodenal ulcer, gastric cancer, and gastric mucosa-associated lymphoid tissue (MALT) lymphoma [8,9].

A vigorous immune response causing inflammation in the gastric mucosa is observed, and the inability to eradicate the organism from the stomach where it leads to persistent infection plays a decisive role in the development of gastric cancer [10]. However, it is a long-term process that takes several decades and is influenced by the gastric environment, host factors, and bacterial virulence factors, resulting in the development of gastric complications such as peptic ulcer disease and gastric cancer [11]. Several *H. pylori* virulence factors that have been estimated to contribute to the pathogenicity and development of gastric cancer are vacuolating cytotoxin A (VacA) (Reviewed by Ansari and Yamaoka [12]), cytotoxin-associated gene pathogenicity island (*cag*PAI), an oncoprotein (i.e., cytotoxin-associated gene A (CagA)), and adhesion proteins [13]. This review was conducted to compile the latest findings regarding CagA and its association with gastric complications development to give a concise report on different aspects of this protein such as its synthesis, transportation, and effector functions. A PubMed search was conducted using the key words “pylori” and “CagA” together or in combination with one more key word such as “pathogenicity,” “gastric complications,” or “gastric cancer.” Among the results, we considered the full-text articles published in English in 2015 or later. However, for greater clarity and deeper insight, we also considered a few older articles. Abstracts, case reports, editorials, commentaries, or manuscripts published in languages other than English were excluded.

## 2. Cytotoxin-Associated Gene Pathogenicity Island (*cag*PAI)

**Figure 1:**
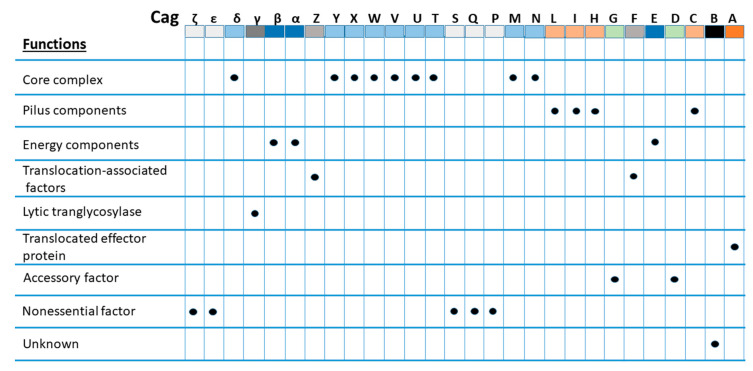
Function of different Cag proteins. The respective function of the Cag protein component is shown by a black circle [16,18].

Nearly 70% of all *H. pylori* strains isolated worldwide have been found to possess *cag*PAI, compared with 95% of East-Asian isolates and 60% of western isolates [13,14]. *cag*PAI is comprised of approximately 40 kb of chromosomal DNA consisting of up to 32 open reading frames (ORFs), namely *cag1* to *cag26*, *cagA* to *cagZ*, or *cagα* to *cagζ*, or by locus name of the HP 26,695 or HP J99 strain genomes [15] that encode effector protein CagA and components of the bacterial type IV secretion system (T4SS), which forms a syringe-like structure to deliver CagA into gastric epithelial cells [16,17]. However, at least 17 genes of *cag*PAI must be expressed to encode intact T4SS with its essential functions, and 14 genes must be expressed to fully induce interleukin-8 (IL-8) secretion [16,18]. It has been suggested that intact T4SS consists of a core complex comprised of CagT, CagX, CagM, Cagδ, and CagY, with associated factors CagH, CagN, CagU, CagV, and CagW; pilus components CagC, CagH, CagI, CagL, and CagY; and energetic components CagE, Cagα, and Cagβ. In addition, CagF, CagZ, and Cagβ act as translocation-associated factors, whereas Cagγ acts as a lytic trans-glycosylase [16], as shown in Figure 1. Inactivation of some of these essential genes has been found to result in a nonfunctional T4SS in strains, rendering no CagA-mediated pathogenicity, which behave like *cag*PAI, completely absent [19,20,21]. Although *cag*PAI integrity is critical to encode intact T4SS for the interaction of *H. pylori* with the host cells, not all strains contain a *cag*PAI, and in some strains, it is incomplete [19,20]. The development of severe gastric complications, such as chronic gastritis, peptic ulcer diseases, and gastric cancer, has been associated with infection with strains expressing the intact *cag*PAI [13,22].

A higher risk of gastric cancer or peptic ulcer disease development has been found in individuals infected with strains harboring *cag*PAI compared with individuals infected with *cag*PAI-negative *H. pylori* strains [13,23]. In a recent study conducted in 263 patients in Alaska, the presence of intact *cag*PAI was detected in 150 (57%) of *H. pylori* strains that were found to be associated with the development of more severe gastric pathology. Out of 12 *H. pylori* strains isolated from gastric cancer patients, 10 (83%) had intact *cag*PAI, whereas 2 strains (17%) had partially deleted *cag*PAI, and the complete absence of *cag*PAI was not found in any of the gastric cancer strains [24]. Similarly, another study conducted in Bulgaria that recruited 156 patients also found the presence of intact *cag*PAI in 100 (64.1%) isolates. In the study, out of 33 strains recovered from peptic ulcer disease (PUD) patients, 29 (88%) isolates had intact *cag*PAI, only 4 (12%) isolates were found to have partially deleted *cag*PAI, and none had completely deleted *cag*PAI genes [25].

## 3. Cytotoxin-Associated Gene A (CagA)

CagA is a 125–145 kDa effector protein, and the strains expressing this protein are considered highly virulent strains, whereas the strains that lack this protein are less virulent *cag*PAI-negative strains [20]. After its synthesis, CagA is translocated into the gastric epithelial cells via T4SS. However, a relatively low amount of it is translocated inside the host epithelial cells despite its abundant synthesis [26].

CagA consists of a well-characterized motif comprising five amino acids, i.e., EPIYA (glutamic acid-proline-isoleucine-tyrosine-alanine), forming a sequence at the C-terminal region, which, together with the neighboring sequence, is responsible for the biological activity of CagA [13]. Depending on the geographic variation, *H. pylori* strains can possess four different types of EPIYA-sequences, i.e., EPIYA-A, -B, -C, and -D. The strains isolated from western countries usually possess CagA with EPIYA-A, -B, and -C (one to three EPIYA-C regions), whereas strains from East Asian countries possess EPIYA-A, -B, and -D sequences. Almost all CagA strains possess the first two EPIYA-sequences, i.e., EPIYA-A and EPIYA-B, whereas the type and presence of the third EPIYA sequence depends on geographic, genotypic, and virulence characteristics, i.e., the EPIYA-C sequence is mostly found in CagA isolated from *H. pylori* in western countries, and the EPIYA-D sequence is mostly from East Asian *H. pylori* [27]. CagA with the EPIYA-D sequence has a higher capability of deregulation of cellular processes than CagA with the EPIYA-C sequence. Therefore, the strains possessing CagA with the first two EPIYA-sequences (EPIYA-A and EPIYA-B) together with the EPIYA-D sequence are considered more virulent than the strains possessing EPIYA-A, EPIYA-B, and EPIYA-C sequences [27]. Studies have also reported an increasing level of virulence with an increasing number of EPIYA-C sequences. A recent meta-analysis found a 1.91-fold increased risk for gastric cancer development associated with the presence of a single EPIYA-D sequence (EPIYA-A, -B, -D) in Asian countries compared with the presence of a single EPIYA-C sequence (EPIYA-A, -B, -C), and the presence of CagA with two or more EPIYA-C sequences (EPIYA-A, -B, -C, -C or EPIYA-A, -B, -C, -C, -C) was associated with a significant increase in the risk for PUD in Asian countries, while a 3.28-fold increased risk was observed for gastric cancer in the USA and Europe [28]. Despite the role of such neighboring sequences of EPIYA-motifs, the amino acid variation in the EPIYA-B tyrosine phosphorylation motif itself has been found to reduce the risk of developing severe complications. It was found that the strains with amino acid polymorphisms within the western-specific EPIYA-B motif, such as EPIYT-B, were found to influence the CagA activity, which reduces the ability to induce the hummingbird phenotype and IL-8 expression, conferring a high risk for duodenal ulcer but a lower risk for gastric cancer development [29].

Several polymorphisms in CagA other than the EPIYA-motifs have been associated with the development of gastric complications. In a recent study, polymorphisms on the nucleotide level in *cagA*, such as *cagA*1283 and *cagA*2551, have been associated with high-grade premalignant lesions, and the polymorphisms such as *cagA*2419 and *cagA*3435 together with polymorphism in *cagL*, such as *cagL*400, have been associated with the risk of gastric cancer development [30]. Moreover, amino acid variations in CagA such as V52I, S194F, and Q/R427K have been associated with the risk of intestinal metaplasia, whereas N467G has been associated with the development of gastric cancer [31]. The studies have found the development of gastric cancer only in 1–2% of *H. pylori*-infected individuals and MALT lymphoma in even less than 0.1% of individuals [32,33]. Translocation of CagA has also been indicated in human B-lymphocytes, leading to the inhibition of apoptosis and continuous proliferation of B-lymphoid cells [34,35]. However, the detailed mechanisms of CagA-mediated pathogenicity still remain unknown. In a recent study, we reported the dominant presence of asparagine (N), serine (S), valine (V), and serine (S) at CagA site 314, 594, 684, and 1077, respectively, in strains isolated from gastric cancer when compared with the presence of amino acids in strain 26695, whereas serine (S), leucine (L), valine (V), and threonine (T) were in the majority of strains isolated from MALT lymphoma patients [36].

## 4. CagA Expression

The pathogenicity of CagA protein results in disturbances in cellular signaling, and the expression of CagA increases gastric cancer risk [37,38]. It was previously shown that a variation in steady-state levels of CagA is found among *H. pylori* strains, modulated by its expression level [39,40]. The expression of CagA is affected by the specific motif. In another study, a high level of CagA expression associated with the strain-specific motif downstream of the *cagA* transcriptional start site (the +59 motif) was found in some strains but not in others [30,40]. This motif (the +59 motif) also stimulated the expression of higher levels of IL-8 production in cultured gastric cells, whereas expression was lower in strains lacking this motif [40]. A recent study reported the significance of this motif together with the neighboring structure. In the study, the +59 AATAAG motif and the adjacent stem-loop structure (stem-loop B) in the *cagA* 5′ untranslated region were found to play an important role in influencing the levels of *cagA* expression [41].

## 5. CagA Translocation

**Figure 2:**
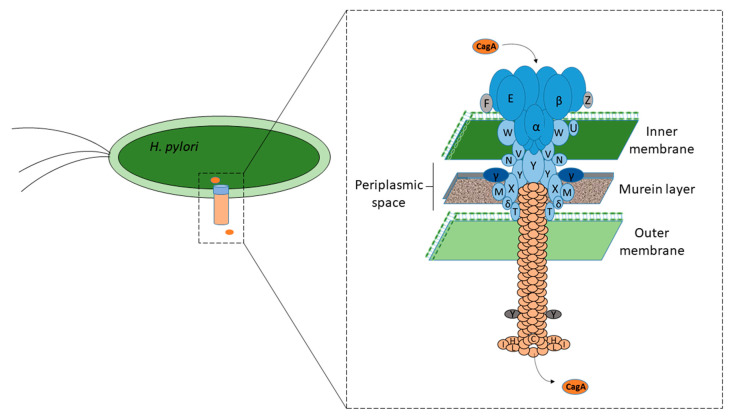
CagA after its synthesis is translocated into the gastric epithelial cells via T4SS, which forms a syringe-like structure [16,18]. The functions of various Cag proteins have been described in Figure 1.

CagA, after its synthesis by *H. pylori* strains, is translocated into the gastric epithelial cells, and at least 15 *cag*PAI-encoded proteins are involved in forming T4SS [16,18], a syringe-like structure (Figure 2). The ultrastructural cytochemical data of a recent in vivo study conducted by Necchi et al., also support the direct translocation of CagA into the gastric epithelial cells by *H. pylori* strains adhered on the lateral plasma membrane [42]. According to a recent study, the protease, i.e., high-temperature requirement protein A (HtrA), produced by *H. pylori* digests the E-cadherin, which acts as the cell adhesion molecule with the adjacent epithelial cells. This disruption enables the bacteria to penetrate the space between the epithelial cells and colonize the basolateral surfaces of the gastric cells [43]. The colonizing *H. pylori* bacteria at the basolateral surfaces have been found to express T4SS in higher numbers than those colonizing the apical surfaces. More than 70% of the *H. pylori* colonizing the basolateral surfaces were found to exhibit the T4SS pili, whereas a majority of the strains colonizing the apical surfaces exhibited no T4SS pili at all or few with only one pilus. This finding suggested the increased capability of *H. pylori* to express the T4SS system apparatus when colonizing the integrin-rich basolateral surfaces compared with *H. pylori* colonizing the apical surface [43]. CagA is exposed on the bacterial surface via T4SS and interacts with the phosphatidylserine (PS) patches that are aberrantly externalized because of the *H. pylori* infection and found on the plasma membrane of the host cells. The N-terminal region of the CagA has been found to interact with the PS patches, and the bound CagA is flipped inside, rendering the internalization of the CagA [44]. However, the translocation of CagA mediated by T4SS across the host cell membrane depends on a number of bacterial as well as host co-factors. The findings of a recent study showed that CagV is closely associated with the surface localization of CagA and its translocation across the host plasma membrane [45].

CagL is a pilin-like component of T4SS encoded by the *cagL* gene (*HP0539*), which binds with human integrin β1-containing receptors, such as integrin α5β1 [16,46]. This integrin binding has been found to be mediated by a tripeptide motif of CagL, i.e., arginine-glycine-aspartic acid (RGD) at residues 76–78, together with the RGD helper sequence, which is a neighboring surface-exposed FEANE (phenylalanine-glutamic acid-alanine-asparagine-glutamic acid) motif [47]. In addition to its importance in the translocation of CagA, the RGD-dependent binding of CagL to integrins has also been found to trigger the intracellular signaling pathways that induce the CagA translocation-independent cell pro-inflammatory response [48,49,50]. The particular polymorphisms in CagL have also been associated with severe disease development, indicating its role in the translocation of CagA. In a study, particular polymorphisms upstream of the RGD motif at amino acid residues 58–62, called the CagL hypervariable motif (CagLHM), were correlated with severe disease progression in a geographically dependent manner [51,52]. Recently, studies have also found CagL polymorphisms such as D58, K59, M60, E62, K122, and I134 and novel CagL variants, and shown their association with chronic gastritis and PUD [53,54].

CagT, another T4SS component, has been found to be essential for T4SS activity [55,56,57,58]. The bacterial lipoproteins have been depicted to play a diverse role in the bacterial cell physiology, antimicrobial resistance, and pathogenicity. CagT undergoes N-terminal modification consistent with lipidation and is required for the delivery of the *H. pylori* oncoprotein, i.e., CagA, into human gastric cells [59]. The lipidation of CagT is necessary for CagT stability, and thus, T4SS activity plays an important role in CagA translocation.

CagY has been elucidated to play an important role in T4SS functionality and, thus, disease severity [58,60,61]. A specific region of the *cagY* gene encodes a part of the CagY protein, which has been found to be an important component in epithelial cell binding [62]. A finding of a recent study also supports its role in the translocation of CagA [63]. In the study, the isolates recovered from α-difluoromethylornithine (DFMO)-treated gerbils harbored the mutated *cagY* that exhibited a reduced ability to translocate CagA by T4SS. In addition, CagY has been elucidated to act as a regulator for immune-sensitive molecules that regulate the immune response to promote bacterial persistence and T4SS function [48,64]. Cagζ (Cag1) has been found to be closely associated with the activity of T4SS, CagA translocation, and IL-8 expression [65]. CagQ, which is a membrane protein in T4SS, plays a key role for maintaining CagA expression and CagA-induced apoptotic effects [66]. CagE protein has been found to induce IL-8 secretion, mediating the host cell cytokine rearrangement in infected cells [57]. Moreover, the results of another recent study have indicated the role of CagX, CagY, CagM, CagT, and Cag3 as an outer membrane complex that anchors the outer membrane of *H. pylori* and plays an essential role in the translocation of CagA [58].

Furthermore, the results of several other studies have suggested the important role of carcinoembryonic antigen-related cell adhesion molecule (CEACAM) receptors binding to the *H. pylori* outer membrane protein Q (HopQ) [67,68,69]. HopQ has been shown to bind with surface-exposed CEACAM receptors on host cells, which brings the T4SS-pilus to the optimal position and distance that allows for efficient CagA delivery into the gastric epithelial cells [69,70]. Moreover, a recent study investigated the interactions between *H. pylori* and three different types of oral epithelial cell lines (HN, CAL-27, and BHY). The results of this study confirmed that the CEACAM receptors that are not usually expressed on oral epithelial cells are important for CagA intracellular delivery [71].

After CagA is internalized into the epithelial cells, it employs at least two distinct mechanisms for the association of internalized CagA to the inner wall of the plasma membrane based on the cell polarity [44]. In polarized epithelial cells, CagA is tethered to the inner leaflet of the plasma membrane through its N-terminal region containing the PS-binding regions (residues 1–876), whereas in nonpolarized cells, the internalized CagA is localized to the cytoplasm.

## 6. Tyrosine Phosphorylation

After its delivery into the gastric epithelial cells, the tyrosine (Y) residue of the EPIYA motifs of CagA undergoes phosphorylation by various cellular kinases such as Csk, Src family kinases (SFKs), and c-Abl [72,73,74]. The tyrosine phosphorylation enables CagA to promiscuously interact with the SH2 domain, containing host proteins such as pro-oncogene Src homology 2 phosphatase (SHP2), PI3K, Crk, or the adaptor protein Grb2 [75,76,77,78]. This interaction perturbs their function, causing dysregulation of the various cell signaling pathways like Ras-ERK MAP kinases (Rap1 → B-Raf → Erk) and Wnt-β-signaling, leading to disturbances in the cellular physiology such as cell–cell adhesion, cellular proliferation, IL-8 expression, and cellular elongation [72,73,78]. The SHP2 phosphatase activity is essential for the full activation of the RAS-ERK signaling pathway, which is deregulated by the interaction of CagA with SHP2 [75]. The ERK signaling pathway has been found to play an essential role in the invasion and progression of several human malignant tumors including gastric cancer [79,80]. Recently, several studies have revealed the activation of the ERK pathway by *H. pylori* infection [81,82].

It was shown that SHP2 binds to the EPIYA-D motif with twofold stronger affinity than to the EPIYA-C motif, suggesting a stronger capability of CagA with the EPIYA-D motif for cellular transformations [83]. A recent study has shown the capability of another protein, SHIP2, containing the SH2 domain, to bind with the tyrosine-phosphorylated EPIYA-C or EPIYA-D segments of CagA. In the study, the complex formation between CagA and SHIP2 was found to tether the SHIP2 to the inner leaflet of the plasma membrane, leading to the increase in the level of membranous phosphatidylinositol 3,4-diphosphate PI(3,4)P2, which could strengthen the adherence of the *H. pylori* to the gastric epithelial cells. The *H. pylori* adhering with greater strength could enhance the CagA delivery into the host epithelial cells, thus enhancing the CagA-SHP2 complex formation and the risk for cellular transformation [84].

## 7. CagA-Dependent Mechanisms of Pathogenicity

Infection with *cag*PAI-positive *H. pylori* strains is considered the strongest risk factor for the development of severe gastroduodenal complications; however, the exact mechanism still remains unclear. CagA, after tyrosine phosphorylation, has been demonstrated to cause the cellular pathogenicity seen in *H. pylori* infection via different mechanisms (Table 1).

A recent study has demonstrated an increased migration and proliferation rate of cells transfected with CagA, suggesting its role in promoting the malignant transfection of gastric epithelial cells [85]. Several studies have found an enhanced probability of gastric carcinogenicity and an induction of the epithelial mesenchymal transition (EMT) process caused by CagA-positive *H. pylori* [86,87,88]. Several recent studies have found that the cells that undergo EMT acquire cancer stem cell (CSC) properties, which could induce the tumorigenesis property due to the ability of self-renewal and differentiation into a vast number of cells [89,90,91,92,93,94]. Bessede et al. conducted a study and suggested an essential role of CagA-positive *H. pylori* infection in the generation of cells with CSC properties in several gastric epithelial cell lines including AGS, MKN45, and MKN74 [95]. The induction of *H. pylori* CagA-mediated EMT has been suggested to be initiated via the stabilization of a protein, Snail1, which plays an essential role in carcinogenesis mainly by the reduction in glycogen synthase kinase-3 (GSK-3) activity [96]. In addition, the CagA-positive *H. pylori*-infected cells have been found to express higher mesenchymal markers such as CD44, vimentin, or zinc finger E-box binding homeobox 1 (ZEB1) [95]. A significantly higher expression of Yes-Associated-Protein (YAP) and TAZ (YAP paralog) has also been shown in cancerous gastric tissues with CagA-positive *H. pylori* infection, suggesting its possible role in transformation of epithelial cells [97]. YAP is a component of the Hippo tumor suppressor signaling pathway playing a crucial role in maintaining the proper size of an organ, tissue homeostasis, cell proliferation, and stem cell properties [98,99,100]. CagA-positive *H. pylori* infection has also been correlated with a decrease in E-cadherin, N-cadherin, and Slug levels, which promotes the EMT process.

Gastrokine 1 (GKN1), a gastric tumor suppressor gene, is predominantly expressed in the stomach, which suppresses the carcinogenicity by reducing the cell viability by inhibiting the cell cycle progression and inducing cell apoptosis [101,102]. GKN1 inhibits the invasion and metastasis of gastric cancer cells, and the downregulated expression of GKN1 has been related to the poorer prognosis of intestinal gastric cancer, which has been demonstrated in chronic gastritis and gastric cancer tissues infected with *H. pylori*, suggesting that *H. pylori*-positive gastritis might progress to gastric cancer [103,104,105,106]. The findings of a recent study also demonstrated that the expression of GKN1 could be significantly downregulated in human gastritis and gastric cancer cells by *H. pylori* infection compared to *H. pylori*-negative gastritis cells, suggesting that its downregulation is mediated by the activation of the CagA-induced ERK pathway and AUF1 upregulation during *H. pylori* infection [107].

A recent study by Li et al. demonstrated that the activation of the YAP signaling pathway could be stimulated by CagA, rendering the AGS cells for tumorigenesis. This phenomenon was also supported by the fact that the activation of YAP expression could be enhanced in concert with E-cadherin suppression in *H. pylori*-infected chronic gastritis tissues compared to *H. pylori*-negative tissues [108]. Moreover, the CagA translocation-independent induction of cell pro-inflammatory response has been shown by CagL, which can trigger the intracellular signaling pathways by RGD-dependent binding to integrins [48,49,50].

Apoptosis-stimulating protein of p53 2 (ASPP2), a host tumor suppressor and an important CagA target, has been found to contribute to the survival of CagA-positive *H. pylori* in the lumen of infected gastric organoids [109]. The interaction of CagA with ASPP2 has been shown to disrupt the cellular polarity, which is essential to form the mucosal barrier, a constituent of the first-line defense mechanism against the invading microbes. Remodeling of the whole PAR complex, including the apical, junctional, and basolateral components, is commenced by the delivery of CagA, suggesting the widespread effects on polarity complex proteins, rendering the loss of cell polarity and the EMT-like phenotype promoted by the interaction of CagA with Par1b [109].

A recent study demonstrated that the *H. pylori* virulence factor CagA could influence the Siva1 protein via increasing ubiquitination and proteasomal degradation mediated by the activation of the PI3K/Akt pathway and XIAP E3 ubiquitin ligase [110]. The data also suggested a downregulation of Siva1, causing inhibition of the apoptotic and DNA damage responses induced by *H. pylori*, whereas CagA was found to inhibit the apoptotic cell death promoting the survival of damaged epithelial cells that contributes to gastric tumorigenesis [110].

Although the presence of *cag*PAI has been suggested to play a key role in the *H. pylori*-mediated pathogenicity, the precise mechanism is still not understood. Recently, the expression of leucine-rich repeats and Ig-like domains 1 (Lrig1), a transmembrane protein acting as an intestinal stem/progenitor cell marker, has been found in the *H. pylori cag*PAI acting in a CagA- and CagE-dependent manner [111]. The findings also suggested a significant increase in Lrig1-positive cells in the premalignant lesions, such as atrophic gastritis and intestinal metaplasia in the antrum and corpus, indicating its possible contribution to the ability of *H. pylori* to cause injury and promote stomach carcinogenesis, which was not found in the normal stomach lining.

In response to a sudden temperature increase, bacterial cells promptly induce the expression of a group of proteins knows as heat-shock proteins (HSPs). The HSPs are crucial for cellular protection, survival, and adaptation to adverse environmental conditions [112]. Several studies have demonstrated the role of heat shock protein 1 (HSP1) in stabilizing the persistent infection mediated by CagA [113,114]. These studies have revealed the CagA-mediated downregulation of HSP1 expression in *H. pylori*-infected cells, and this downregulation, in turn, represses the host response, increasing the probability of *H. pylori* escape from the immune response, which enhances the infection establishment [113,114]. *H. pylori* CagA has also been found to promote cell proliferation by altering the cell cycle via regulation of Reg3, which controls the growth and development of tissues and organs [115]. It was found that Reg3 expression mediated by CagA could influence the occurrence and development of gastric carcinogenesis [116].

Caudal type homeobox 1 (CDX1) is a homeobox transcription factor that plays a crucial role in the development and maintenance of the human intestine [117]. Its activation has been reported to promote cell proliferation, invasion/migration, intestinalization of gastric epithelial cells, and stem cell-like phenotype induction, which leads to cancer development and failure of common gastric cancer chemotherapies [117]. An induction of CDX1 expression by secreted *H. pylori* CagA has been demonstrated in a recent study, suggesting that it plays an important role in the development of gastric carcinogenicity [118].

The presence of CSCs has been demonstrated in cancer tissues of several origins, and because of their abilities to self-renew and transform into multiple cell types, is considered to facilitate tumorigenesis [119]. Specific cell-surface markers have been implicated in the development of CSCs, and CD44 is one of the cell-surface markers with several isoforms generated by alternative splicing of its variant exons [120]. The cancer stem-like cells with CD44 variant 9, one of the isoforms that has been shown to enhance metastasis, contribute to the development and recurrence of gastric cancer and failure of cancer chemotherapies [121,122,123,124,125]. The CD44v9-positive cancer stem-like cells have been shown to accumulate and protect CagA from autophagic degradation after its translocation into the gastric epithelial cells, where it causes the reprogramming and de-differentiation of the fully differentiated cells into tissue stem-like precursor cells with CSC properties [95,126,127]. It has been suggested that the presence of the cells with enhanced expression of the capping actin protein of muscle Z-line α-subunit 1 (CAPZA1) in *H. pylori*-infected gastric mucosa increases the risk of gastric carcinogenesis. In a recent study, an enhanced mRNA expression of CD44v9 was found, which was thought to be induced by the CAPZA1-overexpressing cells with accumulated CagA inside due to the *H. pylori* infection, suggesting the role of CAPZA1 overexpression in the predisposition of cells to develop into CD44v9 cell-surface marker-positive cells due to the accumulation of CagA [128].

## 8. Conclusions

In this study, we indicated the importance of *H. pylori* infection and the role of effector protein CagA in gastric disease development. The strains with intact *cag*PAI mediating efficient CagA translocation are important for the commencement of pathogenicity. CagA, after its translocation and tyrosine phosphorylation, dysregulates the cellular signaling pathways, altering the function of several cellular proteins. Therefore, the CagA-mediated altered activity of several cellular proteins inhibits apoptosis and induces cellular proliferation.

## Figures and Tables

**Table 1:** Mechanisms of CagA-mediated gastric carcinogenesis.

Factors Involved	Mechanism	Outcomes	References
Induced EMT process	Cells undergoing EMT acquire cancer stem cell (CSC) properties	Enhances tumorigenesis	[89,90,91,92,93,94]
	Snail1 protein reduces the glycogen synthase kinase-3 (GSK-3) activity	Enhances carcinogenesis	[95]
YAP and TAZ	Failure in maintaining the organ size, tissue homeostasis, cell proliferation, and stem cell properties	Transformation of epithelial cells	[97,98,99,100]
Gastrokine 1 (GKN1)	CagA-induced activation of ERK pathway and AUF1 upregulation decreases the expression of GKN1	Induces cell cycle progression and inhibits apoptosis, causing invasion and metastasis of the gastric cancer cell	[103,104,105,106]
Apoptosis-stimulating protein p53 2	In association with CagA, disrupts the cellular polarity, damaging the mucosal barrier	Destroys the first-line defense mechanism causing survival of bacteria	[109]
Siva1 protein	Causes the activation of the PI3K/Akt pathway and XIAP E3 ubiquitin ligase	Tumorigenesis via the inhibition of apoptotic cell death, promoting the survival of damaged epithelial cells	[110]
Lrig1	Precise mechanism is unknown	Promotes gastric carcinogenesis	[111]
Heat shock protein 1 (HSP1)	CagA mediates downregulation of HSP1	Promotes persistent infection	[113,114]
Reg3	In association with CagA, alters the cell cycle, reducing its control on development	Gastric carcinogenesis	[115,116]
Caudal type homeobox 1 (CDX1)	CagA induces expression of CDX1, which promotes the cell proliferation and replacement of gastric epithelial cells with intestine-specific cells	Gastric carcinogenesis and failure of common gastric cancer chemotherapies	[117,118]
CD44v9-positive cancer stem-like cells	Protects the accumulated CagA after its translocation from autophagic degradation	Causes the reprogramming and de-differentiation of the cells into cancer progenitor cells	[95,126,127]
